# Fully digital data processing during cardiovascular implantable electronic device follow-up in a high-volume tertiary center

**DOI:** 10.1186/s40001-017-0284-7

**Published:** 2017-10-11

**Authors:** Ingo Staudacher, Asha Roy Nalpathamkalam, Lorenz Uhlmann, Claudius Illg, Sebastian Seehausen, Mohammadreza Akhavanpoor, Anke Buchauer, Nicolas Geis, Patrick Lugenbiel, Patrick A. Schweizer, Panagiotis Xynogalos, Maura M. Zylla, Eberhard Scholz, Edgar Zitron, Hugo A. Katus, Dierk Thomas

**Affiliations:** 1 Department of Cardiology, Medical University Hospital Heidelberg, University of Heidelberg, Im Neuenheimer Feld 410, 69120 Heidelberg, Germany; 2 HCR (Heidelberg Center for Heart Rhythm Disorders), Medical University Hospital Heidelberg, Heidelberg, Germany; 3 Institute of Medical Biometry and Informatics, University Hospital Heidelberg, Heidelberg, Germany; 4 Center for Information Technology and Medical Engineering, University Hospital Heidelberg, Heidelberg, Germany; 5 DZHK (German Centre for Cardiovascular Research), partner site Heidelberg/Mannheim, Heidelberg, Germany; 6 Heidelberg Research Center for Molecular Medicine (HRCMM), Heidelberg, Germany

**Keywords:** Ambulatory device follow-up, Cardiac pacemaker, Digital data processing, Implantable cardioverter defibrillator, Software

## Abstract

**Background:**

Increasing numbers of patients with cardiovascular implantable electronic devices (CIEDs) and limited follow-up capacities highlight unmet challenges in clinical electrophysiology. Integrated software (MediConnect^®^) enabling fully digital processing of device interrogation data has been commercially developed to facilitate follow-up visits. We sought to assess feasibility of fully digital data processing (FDDP) during ambulatory device follow-up in a high-volume tertiary hospital to provide guidance for future users of FDDP software.

**Methods:**

A total of 391 patients (mean age, 70 years) presenting to the outpatient department for routine device follow-up were analyzed (pacemaker, 44%; implantable cardioverter defibrillator, 39%; cardiac resynchronization therapy device, 16%).

**Results:**

Quality of data transfer and follow-up duration were compared between digital (*n* = 265) and manual processing of device data (*n* = 126). Digital data import was successful, complete and correct in 82% of cases when early software versions were used. When using the most recent software version the rate of successful digital data import increased to 100%. Software-based import of interrogation data was complete and without failure in 97% of cases. The mean duration of a follow-up visit did not differ between the two groups (digital 18.7 min vs. manual data transfer 18.2 min).

**Conclusions:**

FDDP software was successfully implemented into the ambulatory follow-up of patients with implanted pacemakers and defibrillators. Digital data import into electronic patient management software was feasible and supported the physician’s workflow. The total duration of follow-up visits comprising technical device interrogation and clinical actions was not affected in the present tertiary center outpatient cohort.

## Background

Cardiac pacemakers, implantable cardioverter defibrillators (ICDs) and cardiac resynchronization therapy (CRT) devices are widely used in clinical electrophysiology [1–4]. The number of cardiovascular implantable electronic devices (CIEDs) is increasing due to aging population and an extension of CIED implantation indications [5] resulting in a rising number of device interrogations. The logistics of monitoring these devices represent a substantial challenge in cardiovascular patient care [6]. As further increase in physician work load cannot be expected in the future, increasing numbers of CIEDs require optimal technical strategies for device follow-up to facilitate device interrogation, data handling, and electronic documentation [7].

During conventional follow-up visits, device data obtained through the programmer are transferred manually into paper-based patient records or electronic patient management systems and device identification documents. To optimize quality and duration of CIED follow-up visits, an integrated software package (MediConnect^®^) has been developed, enabling digital processing of device follow-up data. Fully digital transfer of interrogation data between the programmer and electronic patient management systems is expected to result in increased efficiency with concurrent improvement in reporting quality. Furthermore, the low durability of thermo paper used to print CIED records poses limitation of paper-based follow-up with regard to data storage. Using fully digital solutions, parameters obtained during device interrogation are imported electronically in standardized fashion into the data management system. Written reports and device identification cards are generated automatically by the software. The normalization results in a uniform follow-up process for different devices and manufacturers which may improve workflow productivity.

CIED follow-up in a tertiary center is characterized by high patient numbers, considerable complexity of individual cases, and significant comorbidities of outpatients presenting for routine interrogation. The aim of this work was to evaluate the feasibility of digital CIED data processing using MediConnect^®^ software in a high-volume tertiary hospital.

## Methods

### Study design and patient population

Between November 2013 and November 2015, a total of 18,449 patients presented to the electrophysiology outpatient clinic at the department of cardiology, Medical University Hospital Heidelberg (Germany). A sample cohort of 391 patients scheduled for routine follow-up of cardiac pacemakers, ICDs, or CRT devices were prospectively enrolled. Study patients underwent CIED interrogation, followed by either manual or digital transfer (MediConnect^®^; Fleischhacker, Schwerte, Germany) of device data into the electronic patient management system (i.s.h.med^®^, SAP, Walldorf, Germany) installed on Windows (Microsoft, Redmond, WA, USA)—based personal computer workstations. The decision between manual and digital data processing was left to the physician’s discretion. Baseline characteristics and procedural data were analyzed. Total duration of the follow-up visit was measured from each study subject entering the examination room to the receipt of written medical reports and updated device identification card by the respective patient.

### CIED device interrogation and manual data transfer

**Fig. 1:**
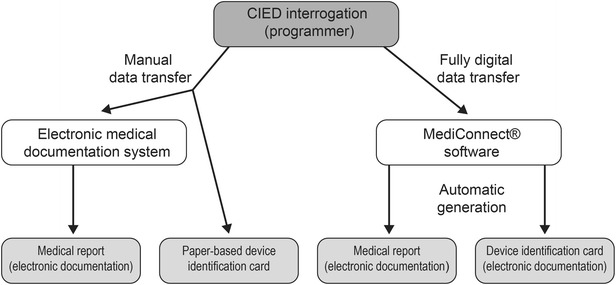
Comparison of manual and digital CIED interrogation data processing during device follow-up

Device interrogation was performed through telemetry (Fig. 1). Specific programmers were used to retrieve programmed parameters and data stored on pacemakers, ICDs and CRT devices from the following manufacturers: Biotronik (Berlin, Germany), Boston Scientific (Marlborough, MA, USA), LivaNova (London, UK), Medtronic (Minneapolis, MN, USA), St. Jude Medical (St. Paul, MN, USA), and Vitatron (Maastricht, the Netherlands). Operating parameters of the devices were modified if required. Interrogation data were then transferred manually into a Microsoft Word-based file of the i.s.h.med^®^ patient management system, allowing for electronic documentation and generation of a medical report. Furthermore, the data were manually entered into a paper-based device identification card. Additional tasks were performed at the physician’s discretion (e.g., obtaining medical history or blood pressure, physical examination, auscultation).

### Fully digital data processing (FDDP)

Following device interrogation via appropriate programming devices, MediConnect^®^ software was employed for digital data transfer and for generation of medical documents. During the course of the study, five versions of the software were used owing to software updates: 2.6.0.130 (10/2013–3/2014), 2.6.0.138 (3/2014–8/2014), 2.7.0.10 (8/2014–10/2014), 2.7.0.20 (10/2014–7/2015), and 2.7.0.47 (7/2015–11/2015). Patients underwent CIED follow-up with the current software version at the time of follow-up. The update from 2.6.0.130 to 2.6.0.138 included minor modifications. More substantial changes were carried out during the following update to version 2.6.0.10, involving optimization of user interface and electrocardiogram viewer. Subsequent updates 2.7.0.20 and 2.7.0.47 were performed to improve stability and performance. The list of programming devices and CIEDs recognized by the software was updated with each step. Interrogation data were stored on a permanently installed USB mass storage stick connected to the programming device. The follow-up data were then imported automatically via a switch box from different programmers employed in parallel into MediConnect^®^ software installed on a personal computer, allowing for standardized reading and storage of data retrieved from different devices (Fig. 1). Medical reports and CIED identification documents were automatically generated by the software package, printed, and stored electronically both in MediConnect^®^ software and through import into the electronic patient management system (i.s.h.med^®^). Thus, the integrated approach allowed for facilitated handling of interrogation data from various manufacturers. Telemedical applications [8] were not included in this work.

### Statistics

Statistical analyses were performed using Prism 6.0 (GraphPad, La Jolla, CA, USA) and Origin 8.0 (OriginLab Corporation, Northampton, MA, USA) software, as well as “R” software [9] (version 3.3.1) with the packages “car” [10] (version 2.1-2) and “xtable” [11] (version 1.8-2). Continuous variables are expressed as mean ± SEM. For between-group comparisons, the unpaired Student’s *t* test (two-tailed test) was used, assuming that the data were approximately normally distributed. Categorical variables are described as count and percentage and were analyzed using the Chi-square test or Fisher’s exact test (two-tailed tests) where appropriate. Furthermore, a linear regression model for the duration of follow-up visits was applied where study group (FDDP vs. conventional follow-up), age, gender, concomitant cardiac disease, device manufacturer, device type, and physician experience in clinical electrophysiology were included as predictors. Effect estimates, 95% confidence intervals (CI), and *p* values are provided in the results section. A *p* value < 0.05 was considered statistically significant. Since this was an exploratory analysis, no adjustment for multiple testing was applied.

## Results

### Patient characteristics

A total of 391 patients presenting for ambulatory follow-up of cardiac pacemakers (*n* = 173; 44%), ICDs (*n* = 154; 39%), or CRT devices (*n* = 64; 16%) were included (Tables 1 and 2). The mean age was 70.3 ± 0.7 years, and 249 study subjects were male (64%). Manual transfer of device interrogation data was employed in 126 cases (32%), whereas fully digital data processing (FDDP) using MediConnect^®^ software was used in 265 patients (68%). The majority of patients exhibited concomitant cardiac disease (manual data transfer, 80%; FDDP, 74%), with ischemic heart disease being the most abundant (Table 1). Sinus rhythm was present in 74% (manual data transmission) and 72% of cases (FDDP). Twenty-three percent of all study subjects was presented in atrial fibrillation. Common CIED indications comprised primary and secondary preventions of sudden cardiac death, higher degree atrioventricular block, and sinus node disease (Table 1). Baseline parameters were not significantly different between groups. Pacemakers and ICDs were equally distributed between the groups (Table 2). Finally, significant intergroup differences between device manufacturers were noted (Table 2).

**Table 1:** Baseline characteristics of study patients

	Manual data processing (*n* = 126)	Fully digital data processing (*n* = 265)	*p* value
Age (years; mean ± SEM)	72 ± 1.1	69 ± 1.0	0.067^a^
Male (*n*; %)	79 (63)	170 (64)	0.78^b^
Concomitant cardiac disease (*n*; %)	101 (80)	197 (74)	0.207^b^
Ischemic heart disease (*n*; %)	66 (52)	120 (45)	0.19^b^
Non-ischemic heart disease (*n*; %)	35 (28)	73 (28)	0.96^b^
Primary electrical heart disease (*n*; %)	0 (0)	4 (2)	0.31^c^
*Rhythm at presentation*			
Sinus rhythm (including complete AV block) (*n*; %)	93 (74)	192 (72)	0.78^b^
Atrial fibrillation^d^ (*n*; %)	29 (23)	59 (22)	0.87^b^
Atrial flutter (*n*; %)	1 (1)	1 (< 1)	0.54^c^
Sinus arrest/complete SA block (*n*; %)	3 (2)	13 (5)	0.29^c^
*Device indications*			
Sinus node disease (*n*; %)	12 (10)	38 (14)	0.18^b^
Atrial fibrillation with slow ventricular response rate (*n*; %)	13 (10)	22 (8)	0.51^b^
AV block (*n*; %)	29 (23)	59 (22)	0.87^b^
Carotid sinus syndrome (*n*; %)	1 (1)	2 (1)	1.0^c^
Primary prevention of SCD (*n*; %)	50 (40)	106 (40)	0.95^b^
Secondary prevention of SCD (*n*; %)	21 (17)	38 (14)	0.55^b^

*AV* atrioventricular node, *SA* sinoatrial node, *SCD* sudden cardiac death, *SEM* standard error of the mean

^a^Unpaired Student’s *t* test (two-tailed test)

^b^Chi-square test (two-tailed test)

^c^Fisher’s exact test (two-tailed test)

^d^Includes paroxysmal, persistent, and permanent atrial fibrillation

**Table 2:** Types and manufacturers of cardiac devices included in the study

	Manual data processing (*n* = 126)	Fully digital data processing (*n* = 265)	*p* value
*Device type*			
Single chamber P (*n*; %)	8 (6)	27 (10)	0.21^a^
Dual chamber P (*n*; %)	43 (34)	95 (36)	0.74^a^
Single chamber ICD (*n*; %)	46 (37)	77 (29)	0.14^a^
Dual chamber ICD (*n*; %)	12 (10)	19 (7)	0.42^a^
CRT-P (*n*; %)	2 (2)	0 (0)	0.10^b^
CRT-D (*n*; %)	15 (12)	47 (18)	0.14^a^
*Device manufacturer*			
Biotronik (*n*; %)	6 (5)	17 (6)	0.52^a^
Boston Scientific (*n*; %)	31 (25)	25 (9)	*< 0.0001* ^a^
LivaNova (Ela/Sorin) (*n*; %)	9 (7)	4 (2)	*0.006* ^b^
Medtronic (*n;* %)	56 (44)	155 (58)	*0.009* ^a^
St. Jude Medical (*n*; %)	22 (17)	63 (24)	0.16^a^
Vitatron (*n*; %)	2 (2)	1 (< 1)	0.24^b^

Italic values indicate statistically significant associations

*CRT* cardiac resynchronization therapy device, *D* defibrillator, *ICD* implantable cardioverter defibrillator, *P* pacemaker

^a^Chi-square test (two-tailed test)

^b^Fisher’s exact test (two-tailed test)

A total of 18 physicians follow-up were involved in the study. Of these, the majority (*n* = 11) applied both conventional follow-up and FDDP. Five physicians used FDDP with MediConnect^®^ software only, and two physicians performed CIED follow-up conventionally in all patients. In the conventional group, 85.7% (108/126) of device interrogations were conducted by investigators routinely using both methods, compared to 86.8% (230/265) in the FDDP cohort (*p* = 0.77). Physician’s experience in clinical electrophysiology differed between treatment groups. Physicians who performed conventional CIED follow-up displayed 3.2 ± 0.2 years of previous electrophysiological experience, whereas in the FDDP cohort, prior experience was lower (1.8 ± 0.1 years; *p* < 0.001).

### Efficiency of digital data transfer

The accuracy of data transfer increased with subsequent software updates (Table 3). Errors included general failure to import interrogation data, incomplete import of single values, insufficient transfer of interrogation values into documents created by the software, and import of incorrect values, respectively. Of note, no errors were observed with the final software update during the course of the study. MediConnect^®^ software crashes occurred during four of 265 follow-up investigations (1.5%). In these cases, a reboot of the system and a re-login were necessary, which required approximately 2–3 min. Two additional system reboots (0.75%) were actively initiated at the physicians’ discretion when document printing failed. Written patient reports and device identification cards produced through MediConnect^®^ software were free from errors in 98% of cases (Table 3). Incompletely transferred device interrogation data were manually completed prior to generation of medical documents, if required.

**Table 3:** Accuracy of data transfer using fully digital data processing, stratified by software versions

	Software version 2.6.0.130–2.7.0.10	Software version 2.7.0.20	Software version 2.7.0.47	All software versions
	(*n* = 38)	(*n* = 210)	(*n* = 17)	(*n* = 265)
General failure of data import (*n*; %)	1 (3)	1 (< 1)	0 (0)	2 (1)
Incomplete data import (one or more values missing) (*n*; %)	2 (5)	3 (1)	0 (0)	5 (2)
Incomplete transfer of values into documents (one or more values missing) (*n*; %)	2 (5)	3 (1)	0 (0)	5 (2)
Import of incorrect values (*n*; %)	2 (5)	0 (0)	0 (0)	2 (1)

**Fig. 2:**
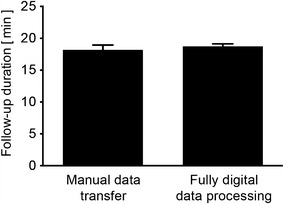
Total duration of ambulatory device follow-up. Mean (± SEM) visit duration is compared between manual and digital data processing, respectively

In the present sample cohort, the mean duration of follow-up visits using FDDP and automatic document production yielded 18.7 ± 0.4 min (*n* = 265) and was not significantly different from patients subjected to manual transfer of interrogation data from the programmer into electronic documentation system and into paper-based device identification cards (18.2 ± 0.8 min; *n* = 126; *p* = 0.49) (Fig. 2). While software updates improved the quality of data transfer, mean follow-up duration did not change when comparing early software versions (16.4 ± 1.0 min; *n* = 38) with the most recent version investigated in the present study (18.4 ± 2.2 min; *n* = 17; *p* = 0.37) (Table 4). Multivariate analysis revealed an influence of physician’s experience and device type on device follow-up duration among potential determinants (i.e., age, gender, concomitant cardiac disease, device type and manufacturer, physician’s experience in clinical electrophysiology, and conventional vs. FDDP follow-up) (Table 5). The duration of ambulatory follow-up was shorter when performed by more experienced physicians.

**Table 4:** Duration of follow-up visits using FDDP software, stratified by software versions

	Software version 2.6.0.130–2.7.0.10	Software version 2.7.0.20	Software version 2.7.0.47
	(*n* = 38)	(*n* = 210)	(*n* = 17)
Mean (± SEM) follow-up duration (min)	16.4 ± 1.0	19.2 ± 0.4	18.4 ± 2.2

**Table 5:** Linear regression analysis of total follow-up

	Regression coefficient	95% confidence interval	*p* value
Intercept	20.21	(15.31; 25.11)	< 0.001
FDDP use (vs. conventional follow-up)	− 0.63	(− 2.22; 0.96)	0.439
Female gender (vs. male)	− 0.82	(− 2.27; 0.64)	0.273
Age (per year)	0.03	(− 0.02; 0.08)	0.261
Concomitant cardiac disease	1.41	(− 0.46; 3.27)	0.141
Physician experience in clinical EP (per year)	− 1.07	(− 1.39; -0.75)	*<* *0.001*
*Device type*			0.009
Single chamber P (vs. CRT-P/CRT-D)	− 2.77	(− 5.83; 0.28)	*
Dual chamber P (vs. CRT-P/CRT-D)	− 1.84	(− 4.11; 0.43)	*
Single chamber ICD (vs. CRT-P/CRT-D)	− 3.49	(− 5.67; − 1.32)	*
Dual chamber ICD (vs. CRT-P/CRT-D)	0.06	(− 2.94; 3.06)	*
*Device manufacturer*			0.243
Boston Scientific (vs. Medtronic)	1.59	(− 0.62; 3.81)	*
Biotronik, LivaNova (Ela/Sorin), Vitatron (vs. Medtronic)	1.20	(− 1.18; 3.59)	*
St. Jude Medical (vs. Medtronic)	− 0.67	(− 2.48; 1.15)	*

Linear regression was applied to adjust for intergroup differences in indicated parameters; * a single *p* value is provided for parameters “device type” and “device manufacturer”

Italic value indicates statistically significant associations

*CRT* cardiac resynchronization therapy device, *D* defibrillator, *EP* electrophysiology, *ICD* implantable cardioverter defibrillator, *P* pacemaker

### Technical and clinical events observed during ambulatory follow-up

In addition to device interrogation, the completion of reports and CIED identification cards, ambulatory CIED follow-up visits included further physician tasks contributing to the duration and complexity of device follow-up. Obtaining patient’s history, physical examination, and consultation with other physicians were commonly performed without significant differences between study groups (Table 6). Furthermore, interrogation of cardiac pacemakers, ICDs, or CRT devices revealed technical issues such as low battery voltage and extended investigation of ICD leads under advisory in a minority of patients of both groups (Table 7). Finally, additional clinical aspects were addressed during device follow-up (Table 8). Ventricular tachycardia or fibrillation episodes detected by defibrillator devices represented the most frequent events and were encountered in 16% (manual data transfer group) and 23% of patients (FDDP; *p* = 0.088). In these cases, further evaluation was necessary and options for antiarrhythmic treatment optimization were discussed and initiated when appropriate.

**Table 6:** Tasks performed by the physician during ambulatory patient follow-up

	Manual data processing (*n* = 126)	Fully digital data processing (*n* = 265)	*p* value
Device interrogation (*n*; %)	126 (100)	265 (100)	1.0^b^
Written report (*n*; %)	126 (100)	265 (100)	1.0^b^
Pacemaker/ICD identification card (*n*; %)	122 (97)^c^	265 (100)	*0.01* ^b^
Brief medical history (*n*; %)	126 (100)	265 (100)	1.0^b^
Consultation with other physicians (*n*; %)	14 (11)	48 (18)	0.08^a^
Patient transferred to emergency department (*n*; %)	1 (1)	0 (0)	0.32^b^

Italic value indicates statistically significant associations

*ICD* implantable cardioverter defibrillator. No patient was directly admitted to the hospital

^a^Chi-square test (two-tailed test)

^b^Fisher’s exact test (two-tailed test)

^c^In five cases, patients were instructed to keep a copy of the written report (including current device parameters) with their pacemaker identification card issued previously

**Table 7:** Device-related technical events identified during patient follow-up

	Manual data processing (*n* = 126)	Fully digital data processing (*n* = 265)	*p* value
Low battery voltage (*n*; %)	6 (5)	6 (2)	0.18^a^
Battery depletion (ERI) (*n*; %)	2 (2)	4 (2)	1.0^b^
Significant change in pacing threshold (*n*; %)	1 (1)	3 (1)	1.0^b^
Significant change in lead impedance (*n*; %)	1 (1)	0 (0)	0.32^b^
Significant change in sensing threshold (*n*; %)	1 (1)	1 (< 1)	0.54^b^
Extended tests owing to lead advisory (*n*; %)	6 (5)	15 (6)	0.71^a^
Lead dysfunction (*n;* %)	0 (0)	1 (< 1)	1.0^b^
Skeletal muscle/nerve stimulation (*n*; %)	0 (0)	0 (0)	1.0^b^
Device-related infection (*n*; %)	0 (0)	0 (0)	1.0^b^
T-wave oversensing (*n*; %)	0 (0)	1 (< 1)	1.0^b^
Pacemaker-mediated tachycardia (*n*; %)	0 (0)	0 (0)	1.0^b^
Cross talk (any type) (*n*; %)	0 (0)	0 (0)	1.0^b^
Electromagnetic interference (*n*; %)	0 (0)	1 (< 1)	1.0^b^

*ERI* elective replacement indicator, *ICD* implantable cardioverter defibrillator

^a^Chi-square test (two-tailed test)

^b^Fisher’s exact test (two-tailed test)

**Table 8:** Clinical events identified during patient follow-up

	Manual data processing (*n* = 126)	Fully digital data processing (*n* = 265)	*p* value
Ventricular tachycardia/fibrillation episodes (*n*; %)	20 (16)	62 (23)	0.088^a^
Ongoing ventricular tachycardia (*n*; %)	0 (0)	0 (0)	1.0^b^
Ongoing supraventricular tachycardia (including AF), requiring immediate attention (*n*; %)	0 (0)	0 (0)	1.0^b^
Change of medication required owing to cardiac conditions (*n*; %)	2 (2)	12 (5)	0.24^b^
Change of medication required owing to non-cardiac conditions (*n*; %)	0 (0)	0 (0)	1.0^b^
In-depth discussion with patient (*n*; %)	9 (7)	11 (4)	0.21^a^
Evaluation of system upgrade from pacemaker to ICD (*n*; %)	0 (0)	0 (0)	1.0^b^
Evaluation of system upgrade from pacemaker/ICD to CRT (*n*; %) (*n*; %)	0 (0)	1 (< 1)	1.0^b^
Evaluation of system downgrade (*n*; %)	0 (0)	0 (0)	1.0^b^

*ICD* implantable cardioverter defibrillator

^a^Chi-square test (two-tailed test)

^b^Fisher’s exact test (two-tailed test)

## Discussion

### Feasibility of fully digital data processing in CIED follow-up

We were able to demonstrate successful implementation of FDDP using MediConnect^®^ software into the clinical CIED follow-up workflow at a university hospital. Feasibility of FDDP in routine application is indicated by a 82% rate of complete and correct data transfer with the first three software versions and a 100% rate of correct data transfer with the final software version which has been used at the end of the study. Software crashes were rare (1.5%). Furthermore, medical documents were produced by the software with very few errors, and provided the added benefit of electronic documentation of both patient reports and device identification cards, respectively, eliminating the use of hardcopy archiving. In addition, removing the need for manual completion of device identification cards resulted in increased technical quality of medical documentation. A low rate of physician complaints after an initial MediConnect^®^ software installation and adaptation phase indicated satisfaction with FDDP performance during the course of the study.

### Comparison with manual data transfer

Optimized data transfer between the programming device and the electronic documentation system could reduce overall duration of follow-up visits. Mean follow-up duration was 19 min in the present study. Contrary to expectations, there was no significant difference between the follow-up duration using FDDP and manual data transmission. Overall times of outpatient visits were determined by additional tasks that were carried out by the physician in addition to device interrogation in the setting of a specialized Electrophysiology Outpatient Clinic, associated with referral of particularly demanding cases. These actions were not affected by the method of handling device-related data. Thus, the relative contribution of efficient data transfer to follow-up times may have been too low to show significant effects. Increased physician’s experience contributed to shorter follow-up duration among patients with manual data handling as well. Furthermore, there was a tendency towards more CRT devices in the FDDP group than in the manual data handling group (18% vs. 13%). As CRT follow-up can be more time-consuming and requires the investigation of more technical parameters compared to pacemakers or ICDs, this difference may potentially have outweighed the benefits of FDDP. Finally, the duration of MediConnect^®^ software loading processes was noted during the course of the study as possible factor contributing to a lack of FDDP effects on total follow-up time. Future software improvements are expected to allow for exploitation of further benefits provided by FDDP use.

### Limitations, benefits and future directions

This study has inherent limitations due to the non-randomized patient assignment to treatment groups. A low recruiting rate resulted in a relatively low number of patients included in the study. It is important to highlight that the subpopulation analyzed here represents only a small “snapshot” of the “real life” situation at the Electrophysiology Outpatient Clinic. In addition, the study was initiated early after new implementation of fully digital data transfer into the clinical workflow. Therefore, benefits of established, long-term FDDP use, expected to result in reduced follow-up duration as device records will be readily available from the database, could not be evaluated. Telemedical applications that may provide significant further improvements in CIED patient follow-up using MediConnect^®^-based digital data transfer were not included in this study. We recognize a potential bias as stratification of the results according to device manufacturers or to the respective physicians performing the follow-up investigations was not statistically feasible in the present sample. However, the open study design and resulting uneven distribution of device manufacturers between manual data handling and FDDP groups at the physicians’ discretion, respectively, may offer further insights into FDDP use. It could be speculated that some manufacturers’ devices were more likely used by the established, manual process, while others were more frequently subjected to be interrogated using the new FDDP workflow algorithm. The hypothesis that FDDP may indeed be better suited for some systems, while other manufacturers’ devices may be associated with potential problems or increased work load, could impact daily clinical routine and needs to be further addressed and evaluated in controlled studies in the future. Potential added benefits of digital data transfer using MediConnect^®^ software were not assessed: Electronic storage of device interrogation and clinical data in the software package facilitates the handling of safety information, device recalls or scientific investigations among CIED patients. While the present study confirms the feasibility of newly implemented digital CIED interrogation data transfer in a high-volume center, a prospective, randomized multicenter study is required to further elucidate characteristics of its long-term use in clinical application.

## Conclusions

Replacing manual CIED data transfer by automatic, fully digital data transfer during routine follow-up is feasible. The overall duration of follow-up visits, including device interrogation, clinical actions, and case discussions, is similar compared with manual data transfer, but may decrease with increasing user experience and further software improvements. Digital data transfer software represents a technical solution to alleviate the growing demand in device follow-up caused by increasing numbers of CIEDs and offers advantages over manual data transfer and paper-based patient records.
